# Supplementary material for: Complex‐centric proteome profiling by SEC‐SWATH‐MS
Source: Mol Syst Biol. 2019 Jan 14;15(1):e8438. doi: 10.15252/msb.20188438 (PMC6346213; doi:10.15252/msb.20188438)
Supplement: Supplementary file 8 — Dataset EV7 [file MSB-15-e8438-s008.zip › feature_plots_string/P37837.pdf]

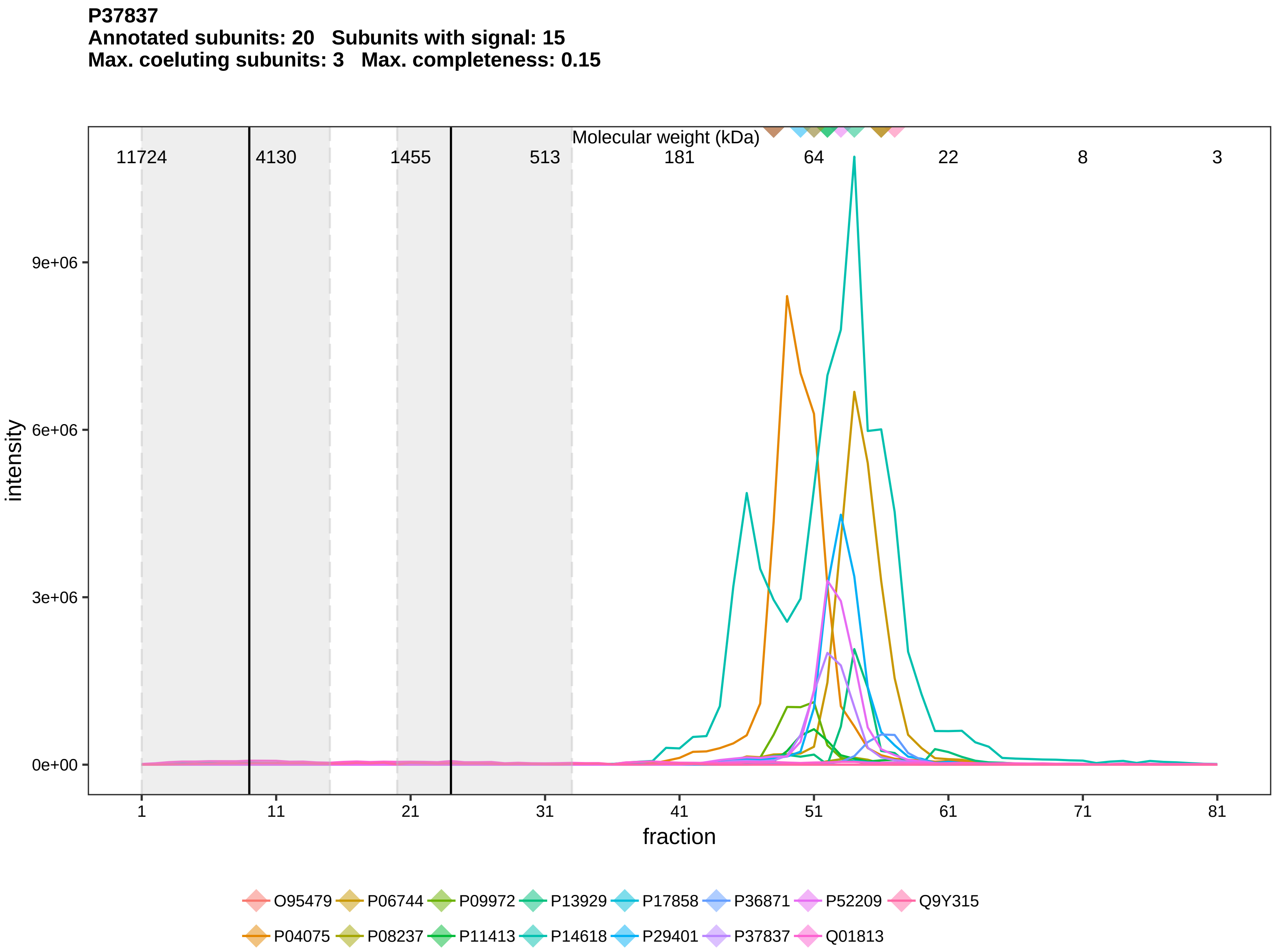
P37837
Annotated subunits: 20 Subunits with signal: 15
Max. coeluting subunits: 3 Max. completeness: 0.15
Molecular weight (kDa)
11724
4130
1455
513
181
64
22
8
3
9e+06
6e+06
3e+06
0e+00
intensity
1
11
21
31
41
51
61
71
81
fraction
O95479
P06744
P09972
P13929
P17858
P36871
P52209
Q9Y315
P04075
P08237
P11413
P14618
P29401
P37837
Q01813
